# Geographic Elevation, Car Driving, and Depression among Elderly Residents in Rural Areas: The Shimane CoHRE Study

**DOI:** 10.3390/ijerph13070738

**Published:** 2016-07-21

**Authors:** Tsuyoshi Hamano, Miwako Takeda, Kristina Sundquist, Toru Nabika

**Affiliations:** 1 Institute of General Education, Kyoto Sangyo University, Motoyama, Kamigamo, Kita-ku, Kyoto 603-8555, Japan; 2 Department of Functional Pathology, School of Medicine, Shimane University, 89-1 Enya-cho, Izumo, Shimane 693-8501, Japan; nabika@med.shimane-u.ac.jp; 3 Center for Community-Based Health Research and Education (CoHRE), Organization for the Promotion of Project Research, Shimane University, 223-8 Enya-cho, Izumo, Shimane 693-8501, Japan; cohre1@med.shimane-u.ac.jp; 4 Center for Primary Health Care Research, Lund University, Clinical Research Centre (CRC), Building 28, floor 11, Jan Waldenströms Gata 35, Skåne University Hospital, Malmö SE-205 02, Sweden; kristina.sundquist@med.lu.se; 5 Stanford Prevention Research Center, Stanford University, Medical School Office Building (MSOB), 251 Campus Drive MC 5411, Stanford, CA 94305, USA

**Keywords:** depression, elevation, rural area, elderly people

## Abstract

Given that public transportation networks are often worse in rural areas than in urban areas, it is difficult for elderly non-drivers to access health-promoting goods, services, and resources related to mental health. Moreover, geographical location, assessed by elevation, could modify this association in a rural area. The aim of this study was to test whether the association between car driving (being a driver or not) and depression, as measured by the Zung Self-Rating Depression Scale (SDS), varied by elevation. Data were collected from a cross-sectional study conducted in the town of Ohnan located in a rural area of Japan. After excluding participants with missing data (*n* = 26), 876 participants were analysed in this study. After adjustment for potential confounders, being a non-driver had a significantly higher odds ratio of SDS (40+) among elderly people living at a low elevation (odds ratio = 2.17, 95% confidence interval = 1.28–3.71). However, similar findings were not observed among elderly people living at a high elevation. These results suggest that car driving importantly predicts depression in elderly people living at relatively low elevations in rural areas.

## 1. Introduction

Depression is a significant contributor to the global burden of disease, and the world mental health survey conducted in 17 countries found that, on average, about 1 in 20 people reported having an episode of depression in the previous year [1]. Several risk factors have been indicated, including socio-economic factors (e.g., educational attainment), physical activity, and other health-related factors (e.g., activities of daily living, and current history of the disease) [2,3,4]. It is also important to note that car driving could be considered a potential risk factor for depression in rural areas [5].

Given that public transportation networks are often worse in rural areas than in urban areas, elderly residents who do not drive can find it difficult to access health-promoting goods, services, and resources [5,6,7]. We therefore hypothesised that elderly people who do not drive are more likely to be depressed than those who are drivers. Moreover, geographical location, as assessed by elevation, could modify the association between car driving (i.e., being a driver or not) and depression. Hilly and mountainous areas occupy approximately 70% of Japan and are defined as the regions extending from the outer plains to the mountains [8]. Health-promoting goods, services, and resources are substantially limited at higher elevations as compared with their availability at lower elevations. Thus, we hypothesised that the association between car driving and depression is stronger in high elevations compared to low elevations.

To the best of our knowledge, no previous studies have investigated potential associations between depression, car driving, and elevation among elderly residents of rural areas. The aim of this study was to test whether the association between car driving and depression varied by elevation in rural areas.

## 2. Materials and Methods

### 2.1. Study Design

Data were collected from a cross-sectional study conducted in 2012. The present study was a part of the Shimane CoHRE Study, which was designed to examine the determinants of lifestyle-related diseases, including depression [9,10,11,12,13]. The Shimane CoHRE study was conducted by Shimane University, Japan, in collaboration with a health examination programme that involved the town of Ohnan. This town is located in a rural area in the southern part of Shimane Prefecture, Japan. Health examination programmes are available once a year for individuals between 40 and 74 years of age who are covered by National Health Insurance. The residents in this town have two options when they wish to undergo a health examination. They may participate in a group examination conducted at public health centres, or they may receive an individual examination conducted at medical institutions. We were permitted to use data from group examinations for the analysis. After excluding participants with missing data (26 participants), we analyzed data from 876 participants. The study protocol was approved by the Ethics Committee of Shimane University School of Medicine in 2010 (number 619). Written informed consent was obtained from all participants.

### 2.2. Self-Rating Depression Scale

The Zung Self-Rating Depression Scale (SDS) was used to assess depression [14]. The SDS is a 20-item self-report questionnaire that is widely used as a screening tool. Each item is scored ranging from 1 to 4, and a total score is provided by summing item scores ranging from 20 to 80, with a higher score denoting greater depression. A depression state was indicated by the SDS score 40+ [15].

### 2.3. Elevation

The Geographic Information Systems software (ArcGIS, version 10.0, Environmental Systems Research Institute, Redlands, CA, USA) was employed for database queries and used to estimate elevation based on the individuals’ addresses. The elevation for each participant was assessed using the ArcGIS ready-to-use dataset of digital elevation models. Participants were divided into two groups on the basis of the median value of elevation (median value = 259.0 m).

### 2.4. Other Measures

We considered the following variables in the analysis: age (years, analysed as a continuous variable), gender (male vs. female), body mass index (BMI) (kg/m^2^, analysed as a continuous variable), current smoking (yes vs. no), current alcohol drinking (yes vs. no), physically active (engaged in physical activity regularly = yes vs. not engaged in physical activity regularly = no), use of medication for metabolic disease treatment (medication to reduce blood pressure, medication to reduce blood sugar or insulin injections, and medication to reduce cholesterol level), having enough sleep (yes vs. no), and car driving (driver = yes vs. non-driver = no). Car driving was assessed by the following question: “Do you have a valid driving licence and usually drive a car?” (yes = driver, no = non-driver).

### 2.5. Statistical Analysis

Descriptive statistics were calculated. The chi-squared test and *t*-tests were used to compare characteristics between the high- and low-elevation groups. Multivariable logistic regression models were performed to derive odds ratios (ORs), 95% confidence intervals (95% CIs), and *p*-values. Independent variables were coded as follows: gender (0 = male, 1 = female), current smoking, current alcohol drinking, medication for metabolic disease treatment, regular physical activity, having enough sleep (0 = no, 1 = yes), and car driving/being a driver (0 = yes, 1 = no). *p*-values less than 0.05 were considered statistically significant. All statistical analyses were performed using IBM SPSS Statistics 20 (IBM Corporation, Tokyo, Japan).

## 3. Results

### 3.1. Participant Characteristics

The characteristics of the study participants are shown in Table 1. There were no statistically significant differences between the low- and high-elevation groups in terms of the SDS score (40+) and car driving (i.e., being a driver or not). Furthermore, the low- and high-elevation groups did not differ significantly in terms of any of the following variables: age, gender, current smoking, medication, BMI, and sleep.

The variables of current alcohol drinking and regular physical activity were significantly different between the low- and high-elevation groups.

### 3.2. Results of the Logistic Regression Analysis

First, interaction effect between elevation and car driving has been checked. As a results, interaction effect between elevation and car driving on OR of SDS score (40+) was statistically significant (*p* = 0.041).

Next, we examined the potential association between car driving and depression as stratified by elevation. Table 2 shows the results of the multivariable logistic regression analysis. For the low-elevation group, being a non-driver had a significantly higher OR of SDS score (40+) (OR = 2.17, 95% CI = 1.28–3.71). In addition, age and having enough sleep were significantly associated with SDS score (40+) (OR = 0.97, 95% CI = 0.94–1.00, and OR = 0.23, 95% CI = 0.14–0.37, respectively).

For the high-elevation group, being a non-driver was not significantly associated with SDS score (40+) (OR = 0.84, 95% CI = 0.45–1.55). The variables of current smoking, regular physical activity, and having enough sleep were significantly associated with SDS score (40+) (OR = 2.07, 95% CI = 1.01–4.25, OR = 0.58, 95% CI = 0.36–0.92, and OR = 0.36, 95% CI = 0.21–0.61, respectively).

## 4. Discussion

To the best of our knowledge, no study has examined the potential association between car driving (i.e., being a driver or not) and depression by considering the effect of elevation in rural areas. Our results showed that the association between car driving and depression varied by elevation: participants who were non-drivers living at lower elevations had a significantly higher OR of SDS score (40+) (OR = 2.17). This association was not observed among elderly residents living at higher elevations. These results suggest that car driving importantly predicts depression in elderly people living at relatively low elevations in rural areas.

We did not find a similar association in the high-elevation group. Participants lived in rural areas; therefore, non-drivers appeared more vulnerable than drivers to the effects of relatively limited access to health-promoting goods, services, and resources. A possible explanation for these results could be that inequalities in socio-economic status (SES) may more significantly affect mental status in low elevations than in high elevations due to sociocultural differences. A previous research conducted in Japan has observed the SES gradient of depression among community-dwelling elderly [16]. To provide this hypothesis, future research should examine the effects of SES on depression in each elevation.

Although more research is needed to examine reasons for why such an association was shown only in the low-elevation group, these results indicate that health policies might focus on providing more effective health care utilization. For example, promotion of mental health checkups would be considered a priority target for non-drivers living at lower elevations (targeted approach). In addition, a population approach is needed to promote good mental health in higher elevation groups.

In an unstratified regression model, car driving did not have a significant association with SDS score (40+) (OR = 1.44, 95% CI = 0.97–2.12). After being stratified by elevation, the effect of car driving on SDS score (40+) appeared more clearly (OR = 2.17, 95% CI = 1.28–3.71). These results imply that it is important to consider not only individual factors but also residential environmental factors for effective approaches in rural areas. There is, however, little evidence examining the effect of residential environment, such as elevation, as measured by GIS. Our study is the first of its kind, and our findings must be confirmed in other settings to provide more robust evidence in the formulation of efficient health policies.

The present study has several strengths. To our knowledge, this is the first study to examine the association between car driving and depression as stratified by elevation. Furthermore, depression was measured by a validated scale [14]. There are also a number of potential limitations to the current study. First, our data set from group examinations may not be an entirely representative sample. A selection bias caused by non-respondents was present and may have influenced the associations shown in our results. Caution is therefore warranted in over-interpreting these findings. However, the participation rate of the group examinations (37.1%) was higher than that of individual examinations (19.1%), and 43.8% of residents did not receive health examinations in this year. Second, misclassification may have occurred in the self-reported data as a consequence of recall errors. However, we have no reason to believe that this potential bias differed between the residential areas. Third, our data did not allow for the assessment of other important factors related to depression (e.g., SES, familial composition, social support, and stressful events). Fourth, participants were divided into two groups according to the median value of elevation. Future research should examine differences in health behaviours or SES gradient between high- and low-elevation groups to create a more robust criterion. In addition, previous study conducted in china used remoteness to examine the effect of geographic location on hypertension [17]. Future research is also required to examine adequate measure related to health-promoting goods, services, and resources in greater depth. Fifth, future research should test our hypothesis in truly mountainous regions. Because people live in such regions are potentially exposed to the relatively limited access to health services. Finally, the present study used a cross-sectional design that did not allow us to establish temporal ordering or causal relationships.

## 5. Conclusions

Our analysis indicates that car driving is significantly associated with the SDS score (40+) of persons living at lower elevations, but not of those living at higher elevations. Further longitudinal research is needed to confirm these findings and to explore the underlying mechanisms.

## Figures and Tables

**Table 1:** Participant characteristics.

Variables	Low Elevation (*n* = 439)		High Elevation (*n* = 437)		*p-*Value
	Number of Participants	% or Mean (SD)	Number of Participants	% or Mean (SD)	
SDS (40+), %	130	29.6	104	23.8	0.052
Driver, %	329	74.9	343	78.5	0.214
Age, years (SD)	439	65.5 (8.2)	437	65.9 (7.4)	0.513
Gender (male), %	168	38.3	190	43.5	0.117
Current smoking, %	58	13.2	54	12.4	0.705
Current alcohol drinking, %	218	49.7	248	56.8	0.035
Regular physical activity, %	186	42.4	228	52.2	0.004
Medication					
To reduce blood pressure, %	160	36.4	147	33.6	0.384
To reduce blood sugar or insulin injections, %	28	6.4	27	6.2	0.903
To reduce cholesterol level, %	93	21.2	100	22.9	0.544
Body Mass Index, kg/m^2^ (SD)	439	22.7 (3.2)	437	22.6 (2.9)	0.587
Having enough sleep, %	332	75.6	347	79.4	0.180

SD, standard deviation; SDS, Self-Rating Depression Scale.

**Table 2:** Multivariable logistic regression analysis with the SDS as the dependent variable.

Variables	Low Elevation (*n* = 439)			High Elevation (*n* = 437)		
	OR	95% CI	*p-*Value	OR	95% CI	*p-*Value
Age (years)	0.97	0.94–1.00	0.049	1.00	0.97–1.04	0.773
Gender (male vs. female)	1.26	0.73–2.18	0.403	1.02	0.59–1.78	0.923
Body mass index (kg/m^2^)	1.00	0.93–1.07	0.975	1.01	0.93–1.09	0.797
Current smoking (no vs. yes)	1.21	0.60–2.45	0.579	2.07	1.01–4.25	0.047
Current alcohol drinking (no vs. yes)	1.44	0.87–2.39	0.149	0.69	0.42–1.13	0.140
Regular physical activity (no vs. yes)	0.79	0.49–1.26	0.325	0.58	0.36–0.92	0.024
Medication (no vs. yes)						
To reduce blood pressure	1.46	0.87–2.42	0.143	1.15	0.67–1.97	0.608
To reduce blood sugar or insulin injection	0.99	0.38–2.57	0.987	0.97	0.35–2.65	0.958
To reduce cholesterol level	0.91	0.50–1.65	0.763	0.73	0.39–1.34	0.318
Having enough sleep (no vs. yes)	0.23	0.14–0.37	<0.001	0.36	0.21–0.61	<0.001
Driver (yes vs. no)	2.17	1.28–3.71	0.004	0.84	0.45–1.55	0.579

OR, odds ratio; 95% CI, 95% confidence interval; SDS, Self-Rating Depression scale.

## Abbreviations

The following abbreviations are used in this manuscript:

SDS	Self-Rating Depression Scale
OR	Odds Ratio
95% CI	95% confidence interval
